# Can we predict who will develop postoperative hyperkalaemia after parathyroidectomy in dialysis patients with secondary hyperparathyroidism?

**DOI:** 10.1186/s12882-019-1416-9

**Published:** 2019-06-20

**Authors:** Yu-Huan Song, Guang-Yan Cai, Yue-Fei Xiao, Yi-Ping Wang, Song-Tao Yang, Xiang-Mei Chen

**Affiliations:** 1 Department of Nephrology, Aerospace Center Hospital, 15 Yuquan Road, Beijing, 100049 China; 2 Department of Nephrology, National Clinical Research Center for Kidney Diseases, Chinese PLA General Hospital, Chinese PLA Institute of Nephrology, State Key Laboratory of Kidney Diseases, 28 Fuxing Road, Beijing, 100853 China

**Keywords:** Hemodialysis, Secondary hyperparathyroidism, Parathyroidectomy, Postoperative hyperkalemia

## Abstract

**Background:**

Hyperkalaemia occurs frequently in many maintenance haemodialysis (MHD) patients after parathyroidectomy (PTX) with secondary hyperparathyroidism (SHPT). However, the clinical risk factors that predict postoperative hyperkalaemia are uncertain.

**Methods:**

This retrospective cohort study included 90 maintenance haemodialysis patients aged ≥18 years who underwent PTX between April 2011 and April 2016 at Aerospace Center Hospital (Peking University Aerospace School of Clinical Medicine). Pre- and post-PTX surgery venous samples were measured in quadruplicate. We examined univariate associations with demographics, dialysis characteristics, laboratory values and medications. Hyperkalaemia was defined as serum potassium >5.3 mmol/L.

**Results:**

Out of nighty patients, twenty-two (24.4%) developed postoperative hyperkalaemia, of whom sixteen (18.1%) developed hyperkalaemia on postoperative day 3. The univariate analysis showed that weight, dialysis duration, preoperative serum potassium, alkaline phosphate, triglyceride, and postoperative alkaline phosphate were independently associated with hyperkalaemia after parathyroidectomy. The univariate logistic regression model showed that preoperative serum potassium was the only independent factor that could predict hyperkalaemia after parathyroidectomy (odds ratio, 1.59; 95% confidence interval, 1.24-2.05). The optimal cut-off for pre-operative K was 3.9 mmol/L according to the receiver operating characteristic (ROC) curve. A higher incidence of postoperative hyperkalaemia was found in male and younger patients, but the difference was not statistically significant (*p*>0.05).

**Conclusions:**

Pre-operative serum potassium less than 3.9 mmol/L was associated with less hyperkalaemia post-operatively in end-stage renal disease (ESRD) patients undergoing PTX.

## Background

Although the survival of end-stage renal disease (ESRD) patients has remarkably improved in recent years, SHPT is a common disorder in ESRD patients receiving MHD [1]. Highly elevated PTH levels in SHPT patients may result in adverse outcomes, such as mineral and bone disorders and high cardiovascular morbidity and mortality. PTX for SHPT in MHD patients was first performed in 1965. PTX continues to be a valuable option for severe SHPT patients who are refractory to medical therapy. Large observational studies have suggested that PTX can reduce the mortality risk [2, 3]. The number of PTX has been decreased in developed countries. However, PTX is required in patients with cinacalcet resistant to SHPT.

The abrupt reduction in serum PTH after PTX may provoke a series of electrolyte abnormalities, such as hypocalcaemia, hypomagnesaemia, hypophosphataemia, and hyperkalaemia [4, 5]. As a result, these patients should be closely monitored, because the immediate postoperative period is the most likely time frame for clinical manifestations of these electrolyte abnormalities [6]. Hypocalcaemia is the most widely recognized disorder and may produce weakness leading to postoperative respiratory distress [7]. Hyperkalaemia is also a complication that occurs within the first 24 h postoperatively after PTX in MHD patients, although the mechanism is not understood [8]. To control hyperkalaemia is one of important issues peri PTX and should be resolved. Hyperkalaemia occurs in 25% to 80% of patients with MHD undergoing PTX. However, this disorder is under-recognized and may lead to more devastating consequences, especially when patients have high normal or elevated baseline serum potassium levels [9–11]. Rosenkrans reported a case of intraoperative hyperkalaemia and ventricular arrhythmia during PTX, which highlighted the under-recognized alterations in potassium homeostasis associated with PTX and underscored the importance of preoperative optimization [6].

Therefore, this retrospective study investigates how multiple perioperative risk factors can lead to postoperative hyperkalaemia in MHD patients undergoing PTX.

## Methods

We enrolled 90 refractory SHPT patients with ESRD needing regular renal dialysis who prepared to receive PTX in the Peking University Aerospace School of Clinical Medicine between April 2011 and April 2016. One expertise surgeon performed all of the surgeries. The study received informed consent in written format from the patients and underwent the approval process of the Research and Ethics Committee of Aerospace Center Hospital.

All of our refractory SHPT patients received thrice weekly dialysis, and the treatment duration was 4 h. The patients were prescribed haemodialysis through a functioning fistula with a blood flow rate of >230 mL/min. The mean and median of the delivered Kt/V were 1.4 and 1.3, respectively, for the haemodialysis patients. Data included age, sex, dialysis duration, the serum creatinine (sCr), blood urea nitrogen (BUN), parathyroid hormone (PTH), alkaline phosphate (ALP),cholesterol (CH), albumin (Alb), haemoglobin (Hb), magnesium (Mg), potassium (K),sodium (Na), calcium (Ca), bicarbonate (HCO3-), phosphorus (P), triglyceride (TG),serum ferritin (SF), and serum iron (SI) levels and angiotensin-converting enzyme inhibitor or angiotensin receptor blocker (ACEI/ARB) use before and after PTX. Hyperkalaemia was defined as serum K >5.3 mmol/L in our hospital.

Patients was resumed for 3 h heparin-free haemodialysis on the day before PTX surgery and on postoperative day 1. The electrolyte K concentration of the dialysate was 2.0 mEq/L. Four venous blood samples were collected before and after heparin-free dialysis, at the end of PTX surgery and pre-dialysis on postoperative day 3 for laboratory chemistry assessments.

The indications for PTX surgery in our hospital were similar to those reported in other studies [8] and included the following: (1) a persistently high PTH level (>800 pg/ml); (2) detection of enlarged parathyroid glands with more than one gland with diameter ≥1 cm on power-Doppler images, and rich in blood flow. (3) uncontrolled hypercalcaemia and hyperphosphataemia, multiple severe bone deformities or extraskeletal calcifications that refractory to medical treatment even though the PTH level was < 500 pg/ml.

MHD patients were excluded if they met one of the following criteria: (1) primary hyperparathyroidism; (2) subtotal parathyroidectomy; (3) previous parathyroid surgery; and (4) pathological diagnosis in addition to parathyroid hyperplasia.

### Statistical analysis

We performed the statistical analysis using SPSS 22.0 version (SPSS Inc., Chicago, IL, USA). Continuous data were presented as means± SD and categorical variables, as numbers or percentages. Student’s *t* test was used to compare continuous variables and Chi-square test for categorical variables. Univariate logistic regression analysis was performed to assess the associations among several demographic and clinical characteristics with hyperkalaemia. Receiver operating characteristic (ROC) curve analysis was further performed to depict the Area under the ROC curve (AUC) for differentiation between preoperative K and postoperative K levels. The cutoff points for preoperative K and postoperative K were defined according to the highest value for sensitivity plus specificity according to the ROC curve. Two-sided *p* values <0.05 were considered statistically significant.

## Results

### The demographic characteristics of PTX patients are given in Table 1.

Ninety refractory SHPT patients (47 men and 43 women) who underwent PTX were included in the analysis. These patients were aged 19 to 70 years, with a mean age of 47.32±10.86 years. The dialysis duration was 8.48±3.39 years. The PTH levels decreased significantly after surgery (from 2137.60±928.01 pg/ml preoperatively to 117.28±154.67 pg/ml) (*p*<0.05). The mean preoperative serum K level was 4.17±0.67 mmol/L. The highest preoperative serum K level was 5.8 mmol/L. If the preoperative serum K level was >5.50 mmol/L, then the operation was postponed. The preoperative serum potassium levels of six patients were<3.5 mmol/L (the lowest potassium level was 3.2 mmol/L), these patients were not given special treatment. After eating potassium-containing foods, we tested the blood again and the results suggested that blood potassium rises to normal.

**Table 1:** Demographic features of ESRD patients with SHPT undergoing PTX

Value	Total (*n* = 90)	Hyperkalemia group (*n* = 22)	Nonhyperkalemia group (*n* = 68)	P
Age(y)	47.32±10.86	46.36±7.40	47.63±11.80	0.004*
Weight (Kg)	63.23±13.08	68.78±14.90	60.10±10.99	0.02*
Dialysis duration(y)	8.48±3.39	7.67±4.25	9.23±2.28	0.03*
Preoperative BUN	22.73±5.49	22.5±4.87	22.9±6.20	0.38
Preoperative Cr	855.11±261.53	835.20±304.50	873.50±225.90	0.64
Preoperative Na	138.19±2.48	138.10±2.28	138.27±2.75	0.35
Postoperative Na	139.10±2.75	138.33±2.57	139.87±2.80	0.92
Preoperative K	4.17±0.67	4.64±0.65	3.73±0.28	0.006*
Postoperative K	4.94±0.89	6.13±0.72	4.55±0.51	0.000
Preoperative Ca	2.60±0.14	2.51±0.07	2.68±0.14	0.06
Postoperative Ca	2.43±0.16	2.39±0.13	2.48±0.18	0.25
Preoperative P	2.38±0.57	2.61±0.63	2.15±0.41	0.99
Postoperative P	2.27±0.47	2.38±0.46	2.18±0.48	0.91
Preoperative Mg	1.01±0.09	1.02±0.05	0.98±0.06	0.30
Postoperative Mg	1.01±0.09	1.01±0.10	1.00±0.09	0.83
Preoperative ALP	534.20±464.20	613.50±579.70	441.78±328.03	0.007*
Postoperative ALP	552.60±562.60	685.90±725.20	429.50±341.70	0.002*
Preoperative PTH	2137.60±928.01	2209.98±920.79	2070.80±967.03	0.76
Postoperative PTH	117.28±154.67	126.43±178.64	108.83±135.77	0.75
Postoperative HCO_3_^-^	21.83±3.10	22.81±3.30	20.92±2.72	0.89
Preoperative SF	878.56±1136.38	688.85±772.01	1053.68±1402.56	0.10
Preoperative SI	11.92±4.66	12.39±3.52	11.5±5.62	0.07
Preoperative CH	4.30±0.89	4.05±0.66	4.52±1.02	0.32
Preoperative TG	1.59±0.86	1.17±0.35	1.97±1.02	0.02*
Preoperative Alb	38.94±3.34	38.8±3.86	39.09±2.93	0.40
Preoperative Hb	109.2±16.85	104.58±19.97	113.46±12.69	0.08

*p<0.05

The mean postoperative serum K level was 4.94±0.89 mmol/L, and the highest was 7.87 mmol/L. Twenty-two of the 90 patients (24.4%) developed postoperative hyperkalaemia (>5.3 mmol/L) after total parathyroidectomy. If the postoperative serum K was >6.0 mmol/L, then the patient underwent emergency haemodialysis. Another 64 patients (71.2%) had normal postoperative serum potassium levels (3.5-5.3 mmol/L). Four patients (4.4%) had postoperative serum potassium levels <3.5 mmol/L, which were increased to a normal level through their diet.

Sixteen of the 90 patients (18.1%) developed hyperkalaemia on postoperative day 3. The mean pre-dialysis K level on postoperative day 3 was 4.66±0.66 mmol/L. The pre-dialysis K level on postoperative day 3 was lower than that in the postoperative K group (*p*<0.05) (Table 2).

**Table 2:** The comparison of postoperative K and pre-dialysis K on the postoperative-3 day (*p*<0.05)

Value	Postoperative K	pre-dialysis K on the postoperative-3 day	p
serum potassium levels	4.94±0.89	4.66±0.66	0.004

### Univariate comparisons of risk factors associated with postoperative hyperkalaemia (Table 1)

In the univariate comparisons, the average age at dialysis in the group with hyperkalaemia was 46.36±7.40 years old, which was younger than the average age in the non-hyperkalaemia group (47.63±11.80 years old) (*p*=0.004). More males (66.7%) were included in the hyperkalaemia group than in the non-hyperkalaemia group (54.6%), but the relevant differences did not reach statistical significance (*p*=0.09).

The average duration of dialysis was 7.67±4.25 years in the hyperkalaemia group, which was significantly shorter than that in the non-hyperkalaemia group (9.23±2.28 years) (*p*=0.03). The average weight was 68.78±14.90kg in the non-hyperkalaemia group and 60.10±10.99 kg in the hyperkalaemia group, which showed a significant difference between the two groups (*p*=0.02). The average serum potassium level was 3.73±0.28 mmol/L in the non-hyperkalaemia group and 4.64±0.65 mmol/L in the hyperkalaemia group, which showed a significant difference between the two groups (p=0.006). The preoperative and postoperative ALP (613.50±579.70 and 685.9±725.2 mmol/L) were both higher in the hyperkalaemia group than in the non-hyperkalaemia group (441.78±328.03 and 429.5±341.7 mmol/L) (*p*<0.001). The average TG level in the hyperkalaemia group was 1.17±0.35 mmol/L, which was significantly lower than that in the non-hyperkalaemia group (1.97±1.02 mmol/L) (*p*=0.02). The nutritional status (Hb, Alb and BUN) showed a poor tendency only in the hyperkalaemia group, but the difference was not significant (p>0.05). No differences were found in the preoperative or postoperative Ca, P, PTH, Mg, Na, Cr, HCO_3_-, CH, SI and SF levels.

### Univariate logistic regression of risk factors associated with postoperative hyperkalaemia (Table 3)

We performed a univariate logistic regression analysis using factors previously identified from univariate analyses as independent variables, including several demographic, clinical and laboratory factors. As shown in Table 3, age, TG and dialysis duration were associated with lower odds of hyperkalaemia, but the differences had borderline statistical significance. Similarly, male sex, weight, preoperative ALP and postoperative ALP displayed no significant trends for increased odds of hyperkalaemia. Thus, the only variable that was independently associated with postoperative hyperkalaemia in the logistic regression analysis was preoperative K (odds ratio (OR) 1.67, 95% confidence interval (CI) [1.25–2.21]), which showed a positive association with the serum potassium level after parathyroidectomy.

**Table 3:** Univariate logistic regression of risk factors associated with postoperative hyperkalaemia

Value	Univariable (OR[95%CI])
Age(y)	0.99 [0.95-1.03]
Weight (Kg)	0.99 [0.35-4.68]
Male	0.43 [0.16–1.15]
Dialysis during(y)	1.02 [0.88–1.19]
Preoperative K	1.59 [1.24–2.05] *
Preoperative ALP	1.00 [0.99–1.01]
Postoperative ALP	1.00 [0.99–1.01]
Preoperative TG	0.50 [0.24–1.07]

*p<0.05

### ROC curve analysis

**Fig. 1:**
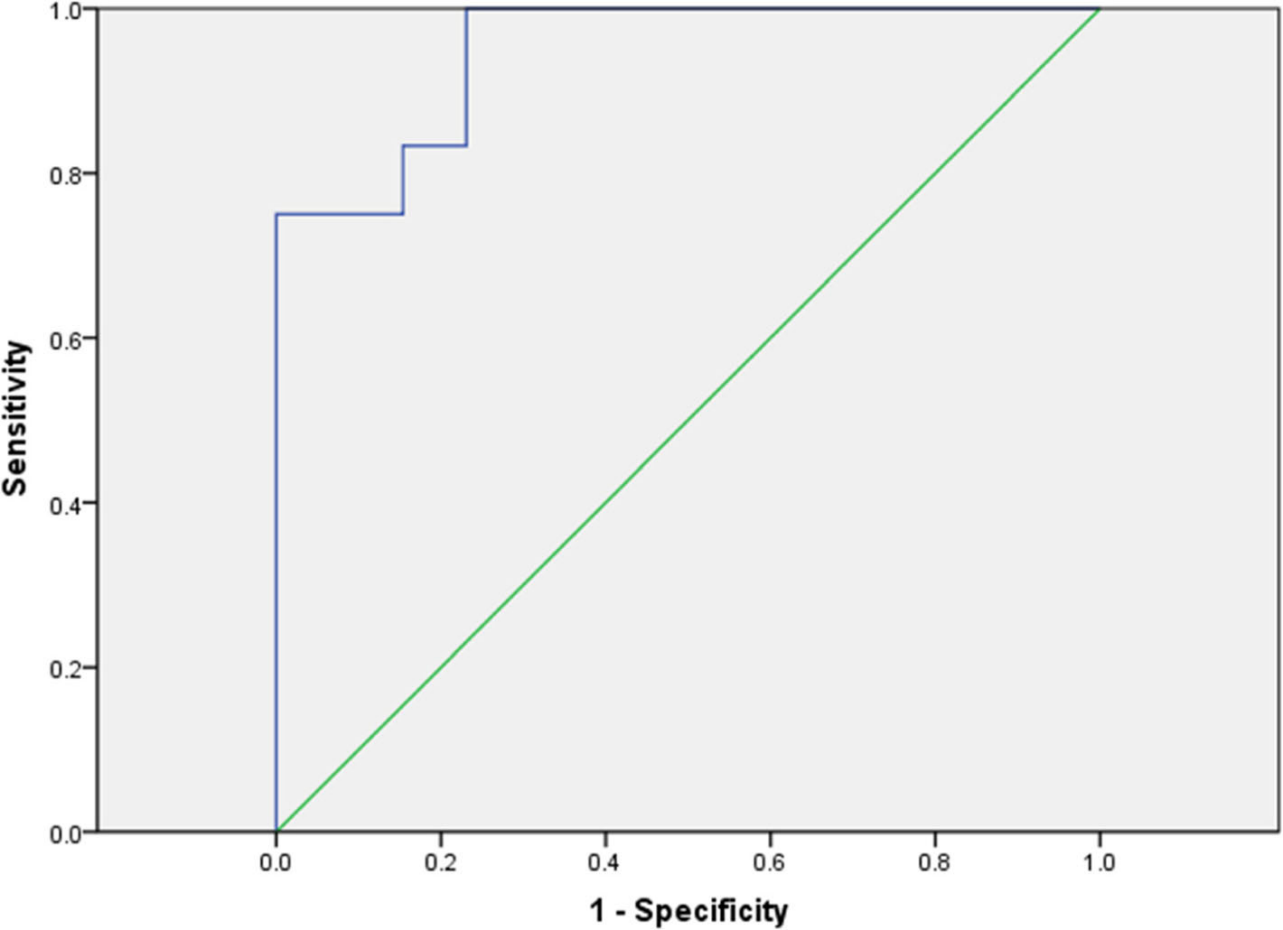
Receiver operating characteristic (ROC) curve of preoperative serum potassium associated with postoperative hyperkalemia

We selected the cutoff value for preoperative K level according to the ROC curve for the early prediction of postoperative hyperkalaemia of refractory SHPT patients received dialysis. The optimal cut-off for pre-operative K was 3.9 mmol/L (sensitivity of 91.7% and specificity of 76.1%, Fig 1). The cutoff point for age was 40.5 years. Unfortunately, due to the low AUC values, we did not obtain cutoff points for ALP, TG and dialysis age.

**Fig. 2:**
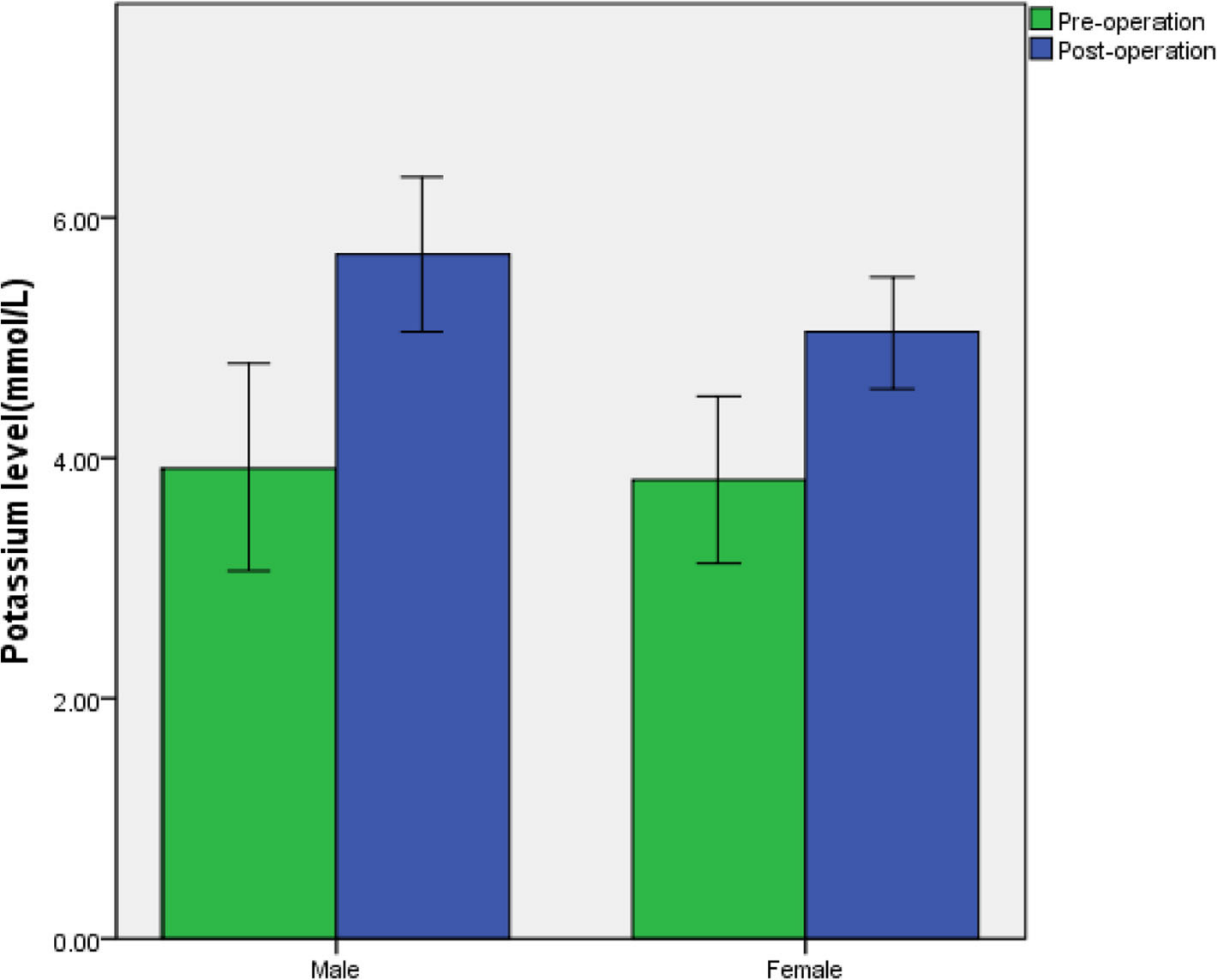
Comparison of the effect of sex on serum potassium levels in ESRD patients undergoing PTX. Data are presented as mean ± SD. *n* = 47 in the female group and *n* = 43 in the male group

Additionally, we compared the effects of sex and use of ARBs/ACEIs on changes in the postoperative potassium levels. Individuals with serum potassium >5.3 mmol/L were slightly more often male (14/43, 32.6%) than female (8/47, 17.0%) compared with the individuals with potassium ≤5.3 mmol/L (Fig 2), but the difference did not reach statistical significance (p=0.09). No difference was found in the use of ARBs/ACEIs between the two groups (p=0.15) (Table 4).

**Table 4:** Variables in ESRD patients with hyperkalemia during PTX

Value	Hyperkalemia group (*n* = 22)	Nonhyperkalemia group (*n* = 68)	P	Odd ratios
Sex				
Female	8	39	0.09	2.96
Male	14	29		
Use of ARB or ACEI				
Yes	9	17	0.15	2.05
No	13	51		
Preoperative K>3.9				
Yes	36	7	0.01*	42.64
No	7	40		

*p<0.05

In Table 5, we summarize the 14 major relevant articles regarding MHD patients with SHPT complicated by hyperkalaemia following PTX. The incidence rate of postoperative hyperkalaemia ranged from 5.3% to 78% [9–20]. Possible risk factors included age, sex, acidosis, hypocalcaemia, surgical trauma, preoperative serum alkaline phosphatase and total calcium supplement dosage, prior treatment with cinacalcet and the surgery duration.

**Table 5:** The basic characteristics of the main literature about hyperkalemia after parathyroidectomy in maintenance hemodialysis Patients

Author	Year	Region	PTX Patients	hyperkalemia Patients	hyperkalemia Patients(%)	Possiblely risk factors
Hayes [9]	1982	Portland	49	9	18.4	hypocalcemia
Shpitz [12]	1986	Israel	--	--	78	hypocalcemia,acidosis
Cruz [4]	1997	USA	1	1	--	hypocalcemia,acidosis
Bajaj [10]	2011	UK	29	16	55	post-induction K^+^ level
Wang [13]	2011	China	135	68	50.4	surgical trauma
Li [14]	2013	China	168	76	45.2	surgical trauma
Yang [11]	2015	Taiwan	25	8	25	acidosis,age,male,surgical trauma
Wu [15]	2015	China	36	6	16.7	--
Song [16]	2016	China	19	1	5.3	--
Chong [17]	2017	Australia	76	17	22.4	Cinacalcet
Pauling [18]	2017	Victoria	22	6	27.3	male,median duration of surgery
Rosenkrans [6]	2017	USA	1	1	--	acidemia, adverse effects of medications, and incomplete dialysis preoperative K^+^ level preoperative K^+^ level, preoperative serum alkaline phosphatase and total calcium supplement dosage
Li [19]	2018	China	108	16	14.8	
Yang [20]	2019	China	204	136	31.9	

## Discussion

SHPT requiring PTX occurs more commonly in patients with progressive chronic kidney disease. Successful PTX often results in a dramatic drop in the parathyroid hormone level, relief of clinical symptoms, and reduced mortality.

There is no agreed upon definition of hyperkalaemia. The above normal limit of the potassium level is 5.3mmol/L in our hospital, and therefore we define hyperkalaemia as a pre-dialysis K>5.3 mmol/L. The European Resuscitation Council recommends stratification of hyperkalaemia into minimal (5.5-5.9mmol/L), moderate (6.0-6.4mmol/L), and severe (≥6.5mmol/L) cases [21]. Due to the high associated mortality, the American Heart Association recommends ‘immediate therapy’ for patients with serum potassium >6.0mmol/L [22].

Hungry bone syndrome (HBS) following PTX surgery is most often associated with hypocalcaemia [23]. However, concomitant hyperkalaemia was also reported in dialysis patients who underwent PTX for SHPT and experienced HBS postoperatively [24]. Is hyperkalaemia a complication of PTX? Fortunately, PTX complicated with hyperkalaemia has attracted the attention of scholars in recent years. One finding suggested that ESRD patients with prior treatment with cinacalcet who were undergoing parathyroidectomy for renal hyperparathyroidism had a greater risk of acute hyperkalaemia during the intraoperative and immediate postoperative periods [17]. Serum K increased quickly since resection of the first hyperplastic parathyroid gland, which accompanied with more parathyroid glands were removed. The decrease of PTH level by parathyroidectomy in SHPT patients exposed cinacalcet can significantly diminish mobilization of skeletal calcium with concomitant hypocalcaemia and hyperkalaemia. Unfortunately, cinacalcet was not widely used until 2018 in China (as a developing country) .Almost none of the 90 patients in our study took cinacalcet. The exact mechanism of this association between hyperkalaemia and cinacalcet is unknown. However, awareness of the phenomenon is imperative for surgeons, anaesthetists and renal physicians.

Shpitz B reported that the mean serum potassium concentration increased from 4.4 mmol/L preoperatively to 6.2 mmol/L within several hours after PTX [12]. A retrospective cohort study of 22 patients who had undergone PTX for SHPT found a 27.3% incidence of hyperkalaemia after PTX. Furthermore, a male dominance of 68% was observed, and the median duration of surgery was longer in the hyperkalaemic patients [18]. A recent publication with larger numbers (204 cases) of PTX patients found that 66.7% patients suffered from hyperkalemia during or immediately after surgery [20].

Many patients routinely (even without surgery) enter dialysis with K >5.3. We needed a comparable control group of patients who did not undergo parathyroidectomy. Therefore, we measured the pre-dialysis K levels on postoperative day 3 and found that the K levels and rates of hyperkalaemia in this group were lower than those in the postoperative K group. Thus, PTX may play a role in the increase in serum potassium.

Our results first indicated that reducing the preoperative potassium levels with preoperative dialysis adequately decreased the risk of postoperative hyperkalaemia in MHD patients undergoing PTX. Preoperative serum potassium concentrations should be controlled below 3.9 mmol/L to avoid postoperative hyperkalaemia.

Additionally, we showed that patients with hyperkalaemia had lower TG levels and a shorter dialysis duration than the non-hyperkalaemia group of patients, presumably due to the patients’ poor compliance. The ALP level was high in the hyperkalaemia group, suggesting that hyperkalaemia might have a relationship with HBS. Cruz found that the rise in the potassium concentration was greater in patients who developed postoperative hypocalcaemia than in patients with normal serum potassium levels and that the highest potassium concentration appeared to be consistent with the lowest calcium concentration [4]. Unfortunately, we did not find statistical significance between the two factors in this study, probably because of the timely regular intravenous calcium supplementations after PTX surgery.

However, the mechanisms underlying the development of postoperative hyperkalaemia following PTX are not clear. A few reports have suggested a causal relationship between hyperkalaemia and HBS. The mechanism has been explained as follows. First, the physiologic calcium pump drives potassium to a location inside the cell membrane. The sharp decrease in the serum calcium levels accompanied by rapid bone absorption after removal of the high PTH levels may trigger a sudden reversal in the physiologic pump mechanism. The kidney of the MHD patient is unable to compensate for this sudden change and develops a high serum potassium level, which may be acute and serious [9]. Second, one study reported that the increase in cellular metabolism might lead to an accumulation of hydrogen and carbon dioxide production. Both of these mechanisms are adversely affected in MHD patients. Finally, PTX patients may manifest life-threatening hyperkalaemia [25]. Third, some anaesthetic drugs can induce life-threatening hyperkalaemia in operative patients, such as succinylcholine and halogenated agents [26]. Mannitol is a type of anaesthesia that usually is used to reduce intracranial pressure. Seto reported that this drug could induce hyperkalaemia and even life-threatening ventricular tachycardia [27]. Furthermore, transfusion of blood products, muscle and surrounding tissue injury, metabolic acidosis, and use of ACEIs/ARBs have all been reported to result in hyperkalaemia [28–30].

Several limitations with regard to this study should be noted. First, parathyroidectomy was the most frequently performed operation in long-term dialysis patients, but there was a broad distribution of many different general surgical procedures, including abdominal or pulmonary resection and orthopaedic or cardiac surgery. The rates of postoperative hyperkalaemia in dialysis patients range from 9.5-24.5% [31–36]. How do the rates of hyperkalaemia compare with those of other surgeries vs PTX? Is the rate of hyperkalaemia higher after PTX than after other surgeries? We cannot draw conclusions from our data and have not found relevant literature reports. Therefore, this issue requires further research. Second, we could not obtain the correlation between postoperative hyperkalaemia and preoperative cinacalcet. Third, this study did not obtain a correlation between postoperative hyperkalaemia and the duration of surgery and did not discover how PTX could lead to specific mechanisms of hyperkalaemia.

## Conclusions

Briefly, this study is a large observational report of how multiple perioperative risk factors affect hyperkalaemia during the immediate postoperative period for ESRD patients who have undergone PTX for renal hyperparathyroidism. Clinicians need to be aware of the risk factors for developing postoperative hyperkalaemia after PTX in MHD patients, especially in male patients younger than 40.5 years. Preoperative severe hyperkalaemia should be monitored, and treatments such as postponing the operation, emergent HD or medication should be considered. Pre-operative serum potassium less than 3.9 mmol/L was associated with less hyperkalaemia post-operatively in end-stage renal disease (ESRD) patients undergoing PTX. This simple scheduling policy is effective at reducing both cost and unnecessary perioperative risks for patients. The relationship between postoperative hyperkalaemia and HBS after PTX needs further study.

## Data Availability

All the data supporting the conclusions of this article are contained within the manuscript. The individual patient-level dataset was not made publically available due to containing potentially identifying patient data; however, the study dataset may be made available from the authors upon request.

## Abbreviations

ACEI/ARB: Angiotensin-converting enzyme inhibitors or angiotensin receptor blockers
Alb: albumin
ALP: Alkalinephosphate
BUN: Blood urea nitrogen
Ca: Calcium
CH: Cholesterol
ESRD: End-stage renal disease
Hb: Hemoglobin
HCO3: bicarbonate
K: potassium
Mg: Magnesium
MHD: Maintenance hemodialysis
Na: sodium
P: Phosphorus
PTH: Parathyroid hormone
PTX: Parathyroidectomy
sCr: Serum creatinine
SF: Serum ferritin
SHPT: Secondary hyperparathyroidism
SI: Serum iron
TG: Triglyceride
